# Impact of adding silver-doped carbon nanotube fillers to heat-cured acrylic denture base on flexural strength, contact angle and surface roughness

**DOI:** 10.1038/s41598-026-48758-y

**Published:** 2026-07-13

**Authors:** Abdulaziz Alhotan, Rasha M. Abdelraouf, Sabry A. El-Korashy, Saleh Alhijji, Hussain D. Alsayed, Jukka P. Matinlinna, Tamer M. Hamdy

**Affiliations:** 1 Department of Dental Health, College of Applied Medical Sciences, King Saud University, P.O. Box 10219, Riyadh, 12372 Saudi Arabia; 2 Biomaterials Department, Faculty of Dentistry, Cairo University, Cairo, 11553 Egypt; 3 Department of Chemistry, Faculty of Science, Suez Canal University, Ismailia, 41511 Egypt; 4 Department of Prosthetic Dental Sciences, College of Dentistry, King Saud University, Riyadh, 12372 Saudi Arabia; 5 Biomaterials Science, Division of Dentistry, Faculty of Biology, Medicine and Health, The University of Manchester, Manchester, M13 9PL UK; 6 Restorative and Dental Materials Department, Oral and Dental Research Institute, National Research Centre (NRC), El Bohouth St, Dokki, 12622 Giza Egypt

**Keywords:** PMMA, flexural strength, contact angle, wettability, surface roughness, Ag-doped carbon nanotubes, heat-cured acrylic resin, Health care, Materials science, Medical research

## Abstract

Acrylic resin, also known as poly (methyl methacrylate) (PMMA), is still the preferred material to fabricate denture bases. Studies were conducted to enhance its mechanical and physical properties. The current in vitro study investigated the effect of adding 0.05 wt% Ag-doped carbon nanotube (CNT) fillers to a heat-cured PMMA-based denture base material on its flexural strength, contact angle, and surface roughness. A total of 60 heat-cured acrylic specimens were prepared. The specimens were divided into two groups (*n* = 30/group), according to the additive used: (a) control group, using heat-cured PMMA; (b) treated group, using an additive powder prepared by mixing 0.05 wt-% Ag-doped CNT nanoparticles with heat-cured PMMA. The flexural strength (three-point bending test), contact angle (sessile drop method), and surface roughness (Microscope Image Analysis Software) for each group were evaluated (*n* = 10/test for each subgroup). Data were analyzed using the independent sample t-test (*p* ≤ 0.05). The flexural strength of the treated group with Ag-doped CNT (107.9 MPa) was significantly higher than that of the control heat-cured PMMA (70.8 MPa). Similarly, the contact angle of the treated group (101.9°) was significantly higher than that of the control group (80.8°). Regarding the average surface roughness (R_a_), no significant difference was observed between the treated group (11.99 R_a_) and the control group (12.42 R_a_). Reinforcing heat-cured acrylic denture base with 0.05 wt-% Ag-doped CNTs enhances flexural strength and hydrophobicity without affecting surface roughness, making it a promising alternative to conventional denture base materials.

## Background

In the dental field, synthetic acrylic polymers are still essential, providing superior biological, and mechanical qualities, which make them perfect for a range of dental applications^1–4^. In dental clinics and labs, poly(methyl methacrylate), (PMMA), remains the preferred material^5,6^. It is used for the construction of a variety of dental appliances such as removable denture bases, occlusal splints for temporomandibular joint therapy, removable orthodontic retainers, interim prostheses, and various surgical splints for craniofacial surgical management^7^.

Perhaps surprisingly, the market does not yet offer any substitutes for PMMA, since the late 1930s. However, it’s crucial to be aware of several of PMMA’s shortcomings, including its poor impact strength, flexural strength, modulus of elasticity, and inadequate surface micro-hardness^8,9^.

Flexural stresses can be initiated by a variety of complicated forces that are applied to a denture in the intraoral environment. Dentures frequently fracture when subjected to impact, flexural, and fatigue stresses because acrylic resin is less resistant to these types of stresses^10^. The long-term use of the denture inside the patient’s mouth can result in fractures due to high levels of flexural stresses from biting. Flexural loading replicates situations in clinics when dentures are subjected to a variety of stresses while in use, such as shear, tensile, and compressive forces^8^. Because conventional PMMA is a brittle material, simple and complex loading frequently results in its failure^11^. Increasing the mechanical qualities of dentures is highly desired.

The surface properties of denture base material is of particular concern, because studies have shown a strong relation between surface roughness and plaque adherence^12,13^. Surface roughness and surface tension have a great influence on the wettability, which can enhance the surface hydrophobicity^14^. A liquid’s wettability on a solid surface is assessed by measuring the contact angle^15^. If the liquid droplet disperses over the surface, leading to a contact angle of under 90°, the surface is hydrophilic. On the other hand, if the water does not spread easily, creating a contact angle of over 90°, the surface is hydrophobic^15^.

Nanotechnology has been advancing dentistry, and a number of studies have been carried out to investigate the possible advantages and ramifications^16,17^. Certain nanoparticles can be added to a polymeric matrix to create nanocomposites^18–20^. The integration of nanoparticle fillers into a PMMA matrix can yield advanced and enhanced mechanical, physical, and antibacterial characteristics, leading to the creation of innovative nanocomposites^21,22^.

Many attempts have been made to enhance PMMA’s mechanical quality in response to the growing need for improved denture base materials, including the use of various fibers, ceramic particles, metallic nanoparticles, or nano-tubes for PMMA reinforcement, exhibiting several advantageous characteristics^16,23–28^.

Silver (Ag) nanoparticles have strong antibacterial effects against common oral pathogens. Moreover, they exhibit a well-tolerated tissue response and low toxicity profile. Therefore, they can be utilized to produce a dental material resistant to bacterial growth and adhesion of plaque^29^, and even as strengthening agents^30^. Adding in a small quantity of Ag-nanoparticles to PMMA composites provides antifungal activity without sacrificing mechanical characteristics^31–33^.

Carbon nanotubes (CNTs) are regarded as one of the most promising nanomaterials with a great deal of potential for biological uses^34^. The goal of integrating nanoparticles and nanotubes is to produce strong, durable, flexible, lightweight, and economically viable nanocomposites^35^. CNTs are already utilized in polymers as reinforcing fillers^36–38^. This could be achieved by facilitating the transfer of stresses from the flexible weaker polymer to the more strong, rigid, and hard nanotubes^39,40^. In addition, dimensional changes of polymers can be minimized by addition of CNTs^36^.

Low concentrations of CNTs can be used as fillers for reinforcement^41^. The CNTs have a high aspect ratio, ultra-light weight, hardness, high tensile strength, superior electrical conductivity, and chemical and thermal stability^42^. The CNTs doped with Ag-nanoparticles (Ag-doped CNTs) might be able to produce special fillers with consistent antibacterial and excellent reinforcing characteristics^43^.

The aim of this in vitro study was to investigate the flexural strength, contact angle, and surface roughness of heat cured acrylic denture base incorporated with 0.5 wt% Ag-doped CNTs nanofillers. The null hypothesis stated that adding 0.5 weight% Ag-doped CNTs to heat-cured PMMA would have no effect on the material’s flexural strength, contact angle, or surface roughness in comparison to the non-treated group (control).

## Methods

The present experimental study was approved by the Medical Research Ethical Committee (MREC) of National Research Centre (NRC), Cairo, Egypt (Ref. number: 163112022). The requirement of informed consent was waived by the MREC of NRC as the current study was *in vitro study*, there is no human participants or sample specimens were taken from any human participants to prepare dentures.

### Sample size calculation

The sample size calculation was based on a similar study and performed using G*Power software version 3.1.9.7 (Heinrich Heine University Düsseldorf, Düsseldorf, Germany), with a significance level of 0.05 and a power of 85% ^24,44,45^. The effect size (f) for flexural strength, the primary outcome, was derived from pilot data (f = 10. 5, based on the difference between the control and treated groups). With this effect size, the minimum required sample size was *n* = 10 per group for testing the flexural strength. Sample size calculations for the secondary outcomes (contact angle and surface roughness) confirmed that the same number of specimens (*n* = 10 per group) was sufficient to detect significant differences.

### Specimens grouping

A total of 60 specimens (*n* = 60) were prepared using poly(methyl methacrylate) heat-cured acrylic resin (Acrostone Heat Cure Acrylic Resin; Acrostone Co.; London; UK). Based on the type of powder employed in the mixing process, the specimens were divided into two main groups (*n* = 30 each group):

- The control group was prepared by mixing heat cured PMMA powder with the monomer liquid.
- The preparation of the treated group involved mixing the heat-cured PMMA powder with 0.05 wt-% Ag-doped carbon nanotube nanoparticles, and then mixing it with the liquid monomer^22^.

### Ag-Doped CNT preparation

Silver nanoparticles were prepared by chemical reduction method by dissolving 50 mL of 0.001 M AgNO_3_ (Silver nitrate; Sigma-Aldrich ChemieGmbH; Schnelldorf; Germany) in bi-distilled water. It was then brought to a boil with a magnetic stirrer on a hot plate. Five mL of 1% trisodium citrate was dropwise added to this solution. The solution was heated until it turned yellowish-brown, as confirmed visually. After that, the heating element was turned off, and it was swirled until it came to room temperature^46^. In order to load Ag nanoparticles into CNT, 0.15 g of multiwalled CNT (CNT Multiwalled; Sigma-Aldrich; Munich; Germany) were gradually added to the solution and vigorously stirred for one hour. The mixture was then filtered by filter paper, rinsed multiple times with distilled water, and dried at 80 °C in a drying oven for twenty-four hours at an inert atmosphere^47,48^.

To create the powder for the treatment group specimens, the heat-cured PMMA powder (0.05 wt-% and 99.95 wt-%, respectively) was combined with Ag-doped CNT and manually mixed in a closed container by shaking it in both horizontal and vertical directions for 10 min to ensure a uniform blend^22^.

### Specimen preparation

#### Wax pattern construction

A stainless-steel mold (64 mm in length, 10 mm in width, and 3.3 mm in thickness) was used to prepare rectangular-shaped wax patterns for the flexural strength test. Moreover, a stainless-steel mold (10 mm diameter × 2 mm height) was used to prepare disc-shaped wax patterns for the contact angle and surface roughness tests. Softened and modified pink wax (Cavex Set Up Regular; Cavex Holland; Haarlem; The Netherlands) was used to fill the molds. The top surface of the mold was covered with a glass slab while the wax was solidifying, while the bottom surface of the wax was contacting the base of the mold. The disc wax patterns were subsequently removed from the mold.

### Flasking and wax elimination

Wax patterns were converted into specimens made of acrylic resin using denture flasks. Following the manufacturer’s directions, gypsum (Gypsona Cast Tape; Gypsano; Cairo; Egypt) and water were combined, and the mixture was then poured into the flask’s lower half. The gypsum was soft, and the wax patterns were embedded in it. Gypsum was left to set. Following the gypsum setting process, the top surfaces of the specimens and gypsum were brushed with a separating material (Acrostone spearing medium; Acrostone Co.; London; UK). Gypsum was prepared and poured over the upper part of the flask after it had been placed. After the flask was filled, a cover was put on top and allowed to solidify fully. For five minutes, the flasks were immersed in 100 °C hot water to extract the wax. After the flasks were opened and the melted wax was taken out, empty molds were left behind.

### Acrylic resin specimen preparation

The PMMA powder and liquid were mixed in a glass container with a 3:1 volume ratio using a stainless-steel spatula. The mixture was filled into the previously prepared mold spaces after it reached the dough stage, and the flask was then painted with a separator. Pressure was applied to the flask until metal-to-metal contact was made followed by wax elimination by immersion in hot water bath (100 °C) for 5 min. In an apparatus for automatic polymerization (Kavo EWL 5501; Kavo Electrotechnisches Werk; Leutkirch; Germany), the discs were polymerized in water at 70 °C for 7 h and then kept at 100 °C for 3 h. When the flask was cured, it was allowed to cool gradually. The specimens were prepared at completion of the de-flasking procedure and removal of any remaining material using an acrylic bur. Sandpaper was used to smooth the discs before polishing them with polishing brushes loaded with pumice. Final fine polishing was not performed to preserve surface characteristics. The discs were rinsed with distilled water. Prior to testing, distilled water was used to ultrasonically clean each disc for 20 min. The discs were then stored at 37 °C in distilled water. ^49,50^.

### Flexural strength test

In accordance with ISO 20795-1, a three-point bending flexural strength test was used to measure the flexural strength^51^. Using stainless steel mold, specimens measuring 64 mm in length, 10 mm in width, and 3.3 mm in thickness were created^51^. The specimens were tested using a universal testing machine (UTM Model 3345, Instron Industrial Products; Norwood; MA; USA) with two supports spaced 50 mm apart and the crosshead speed of 5 mm/min was used, with a load cell of 500 N. The following formula was used to determine the flexural strength (FS) in MPa^28,52^:

$$\rm FS = PL/wb^2$$

Where: =the maximum force at fracture and recorded in Newtons (N), L=distance between supports (20 mm), and b = the thickness and height of the specimen respectively and measured in mm.

### Contact angle test

Disc specimens of 10 mm in diameter and 2 mm in height were prepared using a stainless-steel mold^45^. The contact angle was assessed using the sessile drop method, a droplet analysis equipment (SmartDrop; Femtofab; Seongnam; Korea) by using a 5 µL droplet of H_2_O dropped at a rate of 2.0 µL/s in the center of each specimen surface at room temperature to determine the hydrophilicity of each sample^7,53^. For each specimen, measurements were repeated three times to take its average.

### Surface roughness test

The morphology of the surfaces was investigated using a non-destructive environmental scanning electron microscope, SEM (SEM Quanta 250; FEI Company; Eindhoven; The Netherlands) at 1000× magnification^54,55^. Disc specimens as described above were used^56,57^. Using the imaging analysis system Scandium Solution Height (Scandium Solution; Olympus Soft Imaging Solutions; Hamburg; Germany), SEM images were transformed into third-dimension images. The average surface roughness (R_a_) of each specimen was determined from measurements taken at three different locations. The SEM micrographs were used to evaluate the surface roughness of the same specimens^58–60^.

### Statistical analysis

The statistical analyses were conducted on statistics software (IBM SPSS Statistics for Windows, Version 23.0; IBM Corporation, Armonk; NY; USA). The Kolmogorov-Smirnov and Shapiro-Wilk tests were all non-significant (*p* > 0.05 in all cases). This demonstrated that the distributions were relatively normally distributed. Thus, the flexural strength, contact angle, and surface roughness values were analysed and compared using independent samples t-tests. The conventional value of *p* ≤ 0.05 was adopted as the criterion for statistical significance. Cohen’s d effect sizes (d), a standardized measure of the magnitude of differences between groups, were calculated to quantify the practical significance of the results. Additionally, 95% confidence intervals (CIs) were calculated to assess the precision of the measurements.

## Results

**Fig. 1:**
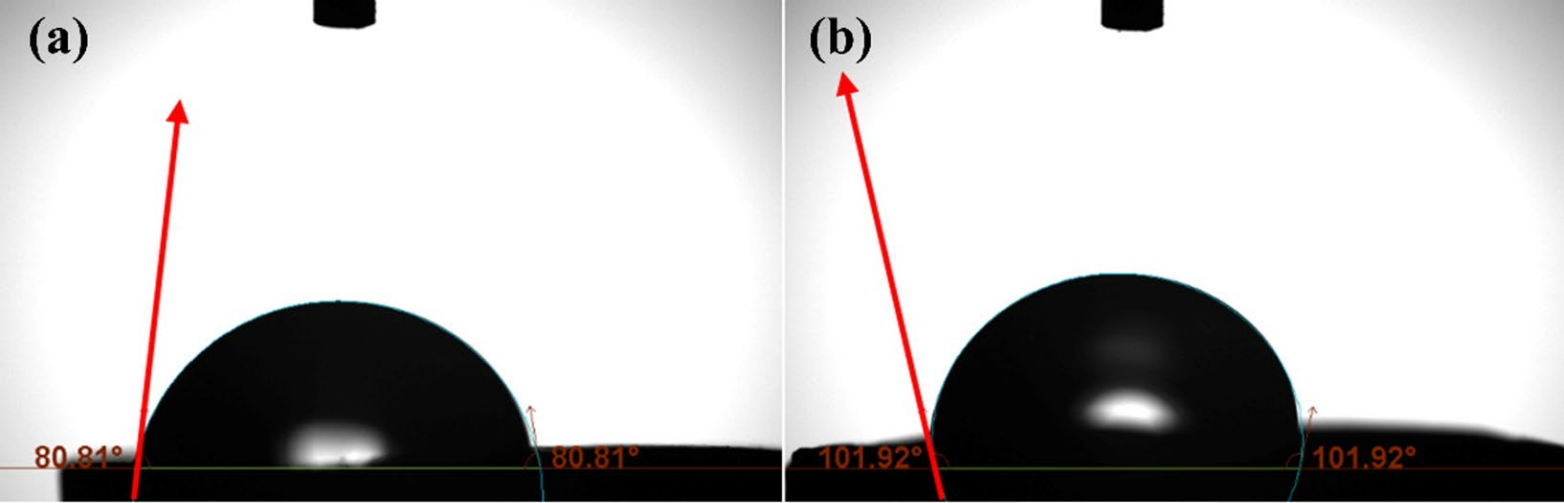
Representative images of water droplets on different groups: (**a**) control group’s lower contact angle=higher wettability=lower hydrophobicity; (**b**) treated group’s higher contact angle =lower wettability= higher hydrophobicity.

**Fig. 2:**
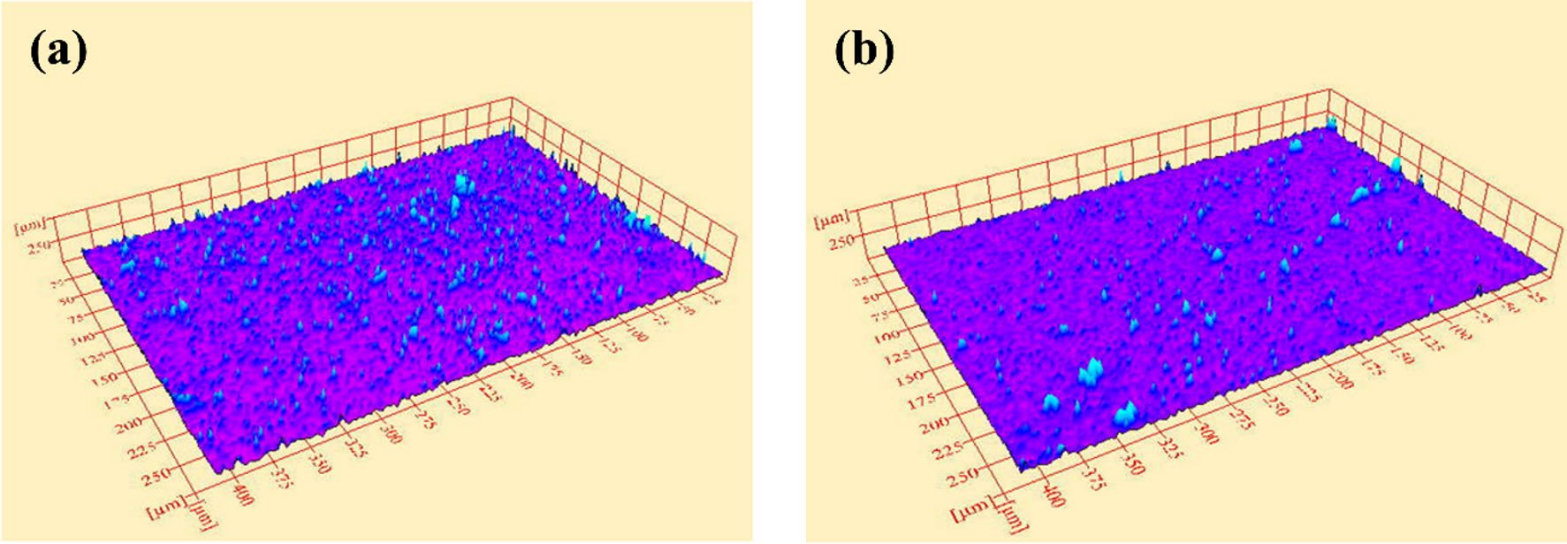
Representative 3-D surface roughness images (R_a_): (**a**) control group, (**b**) treated group.

**Fig. 3:**
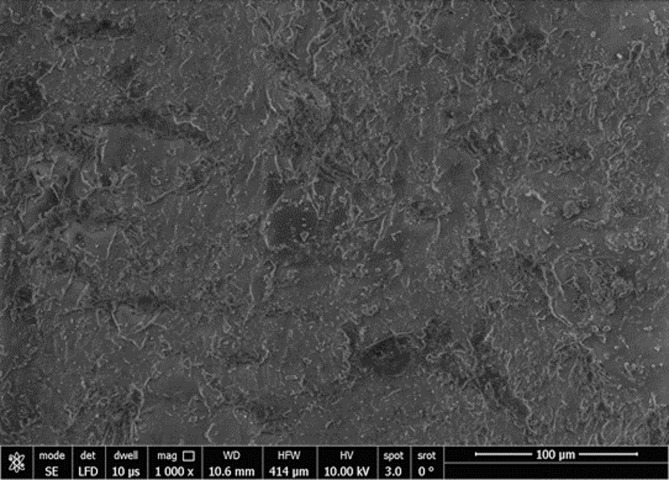
Representative SEM micrograph of the control group at 1000X magnification.

The mean, standard deviation values for flexural strength (MPa), contact angle (°), and SEM surface examination images, 3-D average surface roughness R_a_ (µm) are presented in Table 1; Figs. 1, 2 and 3.

**Table 1:** Mean values of flexural strength (MPa), contact angle (°) surface roughness (R_a_) for both groups.

Test	Control Group	Treated Group	*p* Value (sig.)
Flexural strength (MPa)	70.80 (1.48)	107.90 (1.97)	*p* = 0.001*
Contact angle (°)	80.81 (0.08)	101.92 (0.09)	*p* = 0.001*
Surface roughness (µm)	12.42 (0.11)	11.99 (0.97)	*p* = 0.6

* p-values signify statistical significance between groups (*p* < 0.05).

The results showed a significant increase in flexural strength and contact angle in the treated groups compared to the control group, while no significant difference was observed as regards surface roughness.

### Flexure strength

There was a significant increase in flexural strength of PMMA of the treated group after the addition of Ag-doped CNT (107.90 MPa) compared with the control group (70.80 MPa), (*P* = 0.001*).

Effect size for flexural strength was very large (d = 21.3), indicating that nanoparticle incorporation substantially improved flexural strength, with a narrow confidence interval reflecting high measurement precision (95% CI: 36.18–38.02 MPa).

### Contact angle

The treated group showed a significant higher contact angle values (represent a lower wettability, and more hydrophobicity) (101.92 °) than the control group (80.81 °), (*p* = 0.001*), Fig. 1.

Effect size for contact angle was very large (d = 248.4), indicating that nanoparticle incorporation substantially increased the contact angle (reducing wettability), with a narrow confidence interval reflecting high measurement precision (95% CI: 21.065–21.155°).

### Surface roughness

There was no significant difference in the average surface roughness (R_a_) between the treated group (11.99 μm) and the control group (12.42 μm), (*p* = 0.6), Fig. 2.

Effect size for surface roughness showed no significant change (d = − 0.62), with values ranging within a narrow interval (95% CI: −0.794 to − 0.066 μm), indicating that nanoparticle incorporation did not adversely affect surface topography.

**Fig. 4:**
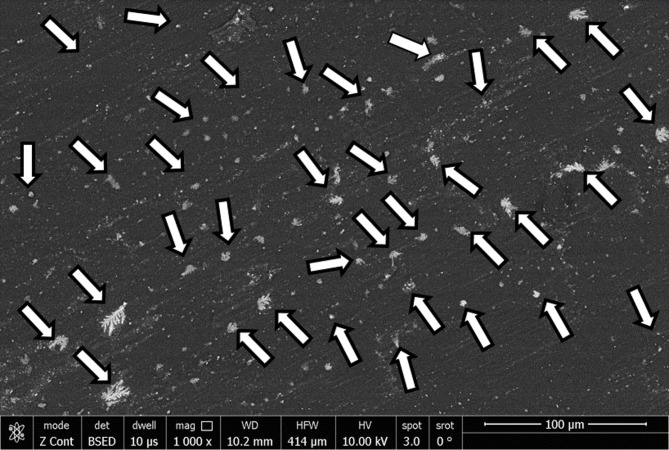
Representative SEM micrograph of the treated group at 1000X magnification. Arrows are directed towards the Ag-doped CNT.

Figures 3 and 4 represent SEM micrographs for the control and treated groups respectively which had been used to produce the 3D images to measure the surface roughness. The arrows in Fig. 4 pointed to the clustered Ag-doped CNT fillers with variable sizes and shapes. The SEM micrograph of the treated group (Fig. 4) displayed the well distribution of the fillers within the polymeric matrix. In contrast, the SEM micrograph of the control group did not contain such fillers (Fig. 3).

## Discussion

The incorporation of carbon nanotubes into some dental materials is anticipated to augment their utilization in dentistry and facilitate novel functional applications due to their enhanced mechanical characteristics^22,37^. These developments along with the addition of Ag-nanoparticles could result in improved mechanical and surface properties along with antibacterial effects^41,42^. The doping method used above can produce a hybrid nanostructure that displays characteristics from both parent components^61^.

The current in vitro study investigated the flexural strength, wettability and surface roughness. The null hypothesis was rejected, as the findings of the current study indicate that adding 0.05 wt-% Ag-doped CNT nanofillers to PMMA produced a novel nanocomposite with higher flexural strength, contact angle which corresponds to lower wettability and higher hydrophobicity, and no effect on surface roughness compared with the control group.

The significant increase in the flexural strength in the treated PMMA groups may be due to the addition of the Ag-doped CNT nanoparticle fillers, which could transfer stresses from the weak polymeric matrix to the stronger filler phase. In addition, these fillers could inhibit the development of cracks inside the polymeric matrix. ^62^. This finding agreed with previous studies which reported an increase in the mechanical properties of PMMA after the addition of CNT^22,63^. The increase in flexural strength align with clinical needs for durability in denture applications.

The increase in the contact angle corresponding to lower wettability and higher hydrophobicity could be explained by the presence of CNT nanoparticles. This may be attributed to the fact that CNTs are by nature hydrophobic and tend to self-aggregate due to the van der Waals forces^64^. In addition, the presence Ag-nanoparticles leads to charge transfer from the Ag-ions to the multiwalled CNTs, which in turn enhance the hydrophobic feature^65^. This is consistent with previous studies that have reported that nanocarbon materials tend to be hydrophobic or superhydrophobic, exhibiting high contact angles that can exceed 165° ^66,67^ Although unblended CNTs may have an inherently hydrophilic nature, the addition of liquid hydrocarbons (such as the methyl methacrylate “MMA” monomer) to the nanocarbon can render the CNTs hydrophobic^67^.This hydrophobicity could aid clinically to reduce biofilm formation or resistance to staining.

The surface roughness was assessed by the microscopic-image-analysis-software to study the area of interest in its effective magnification and produce a comprehensive analysis of the surface. Other tools, such as a stylus profilometer, could scratch the surface and cause its distortion^68^. While the atomic force microscope (AFM) scans only a smaller area and provides data at the nanoscale level, which could not be compared to the clinical critical value that was reported in the literature by microns to accumulate plaque, bacteria, and stains^58,60^.

No significant difference was detected in the surface roughness values between the treated and control groups. This could be explained by the low concentration of the added fillers (0.05 wt%) in the form of nanosized particles, which could agglomerate into clusters which if scratched led to nano-roughness^24^. A previous study investigated the addition of 1.0 wt% CNT to denture base material using a profilometer, and the results showed a reduction in surface roughness after adding the CNT^69^. This was attributed to the small filler size and the presence of only few particles in the surface^70^.

The surface roughness values obtained in this study were higher than those commonly reported for conventionally polished denture base materials and exceeded the clinically accepted threshold of 0.2 μm for plaque accumulation. This may be attributed to the used polishing protocol, in which the final fine polishing step was intentionally omitted to avoid masking the inherent surface characteristics of both the control and treated specimens, thereby allowing direct evaluation of the effect of nanoparticle incorporation on surface roughness.

Although the SEM of the treated group displayed a well distribution of Ag-doped CNTs within the polymeric matrix areas, there was a variation in the size of the fillers due to the random agglomeration and clustering of CNTs^22,71^ as well as the use of manual rather than automated mixing. However, manual mixing was chosen to simulate a simple, clinically feasible method, especially since a low concentration (0.05 wt-% Ag-doped CNTs) and thorough 10-minute mixing were employed. However, manual blending may have limitations, as it can increase the potential for agglomeration and reduce uniform filler distribution. Therefore, alternative techniques such as ultrasonication or mechanical stirring are recommended to standardize mixing and mitigate variability.

Previous research conducted by Alhotan et al. (2023)^21^ as well as the present study both investigated the incorporation of Ag-doped CNTs into PMMA denture base materials. Alhotan et al. (2023)^21^ focused on impact strength, microhardness, and antimicrobial activity, while the current study evaluated flexural strength, surface roughness, and contact angle, which have not previously been reported for this filler system. Together, these studies demonstrate the multifunctional enhancement of PMMA with Ag-doped CNTs and highlight the novel assessment of flexural and surface properties in the present work.

Further studies were suggested to assess aesthetics and any possible color changes. Moreover, the filler concentration and its effect on the long-term material properties, like wear resistance, could be investigated, in addition to its cytotoxicity. Moreover, evaluating various concentrations and types of fillers in PMMA would give a deeper insight into this composite system in a trial to improve its properties.

## Conclusions

The innovatively reinforced heat-cured acrylic denture base with 0.05 wt-% Ag-doped CNT nanofillers had the opportunity to be used as an alternative to conventional heat-cured acrylic denture base as it provides enhanced flexural strength and hydrophobicity without compromising the surface roughness.

### Significance

Reinforcing heat-cured acrylic denture bases with 0.05 wt-% Ag-doped CNT nanofillers improves their flexural strength, reducing the risk of denture base fracture during service and extending durability. Furthermore, the enhanced hydrophobicity reduces the possible water absorption, thereby preventing staining, odor, and microbial adhesion. This could be translated into real-world dental applications by improving denture long-term performance and patient satisfaction.

## Data Availability

The data that support the findings of this study are available from the corresponding author upon reasonable request.

## Abbreviations

PMMA: poly-methyl methacrylate
Ag: silver
CNTs: carbon nanotubes
R_a_: surface roughness
wt-%: weight%
MREC: Medical Research Ethical Committee
NRC: National Research Centre
ISO: International Organization for Standardization
N: Newtons
fs: flexural strength
MPa: megapascal
